# Retromuscular prophylactic mesh reinforcement after midline laparotomy: a systematic review and meta-analysis

**DOI:** 10.1007/s10029-025-03533-2

**Published:** 2025-12-27

**Authors:** Melissa Lagger, Raffaella Sguinzi, Leo Buhler, Michel Adamina

**Affiliations:** 1 Department of Surgery, Cantonal Hospital of Fribourg, Chemin Des Pensionnats 2/6, 1752, Villars-Sur-Glâne, Fribourg, Switzerland; 2 Department of Medical and Surgical Specialties, Faculty of Science and Medicine, University of Fribourg, Fribourg, Switzerland; 3 Department of Visceral Surgery and Medicine, Inselspital, Bern University Hospital, Bern, Switzerland

**Keywords:** Retromuscular mesh reinforcement, Sublay mesh, Incisional hernia prevention, Abdominal wall closure

## Abstract

**Purpose:**

Incisional hernias (IH) are a frequent complication after laparotomy, contributing to patient morbidity and increased healthcare costs. While guidelines recommend prophylactic mesh reinforcement (PMR) in high-risk elective surgeries, there are no specific recommendations for contaminated/emergency settings. The retromuscular plane is considered optimal for mesh placement due to its favourable outcomes. This systematic review and meta-analysis aimed to evaluate the effectiveness and safety of retromuscular PMR in reducing IH rates following elective and contaminated/emergency midline laparotomies.

**Methods:**

Following the PICO framework, we included studies assessing patients undergoing elective or contaminated/emergency midline laparotomies. The intervention was retromuscular PMR compared to primary suture closure (PSC). The primary outcome was IH incidence, with surgical site infection (SSI), seroma, and hematoma as secondary outcomes. A systematic literature search was conducted in Medline, Embase, Web of Science and Cochrane Library, the last search was completed on March 7th, 2025. Risk of bias was assessed using the RoB 2 tool. A random-effects meta-analysis was performed, with subgroup analyses by mesh type and surgical setting.

**Results:**

Eight randomized controlled trials totalizing 1167 patients were included. PMR significantly reduced the risk of incisional hernia at the longest available follow-up compared to primary suture closure (OR 0.37, 95% CI 0.17–0.80), but heterogeneity was high (I^2^ = 74%). Subgroup analysis showed benefit with synthetic mesh (OR 0.18, 95% CI 0.06–0.52) but not with biologic mesh. No significant differences were observed in surgical site infection, while non-significant trends toward increased seroma (OR 1.97) and hematoma (OR 3.05) were noted. Evidence in contaminated/emergency settings was limited and exploratory.

**Conclusion:**

Retromuscular prophylactic mesh reinforcement reduces incisional hernia incidence in elective laparotomy, particularly with synthetic mesh, without increasing major infectious complications. However, substantial heterogeneity across studies limits the certainty of effect estimates, and evidence in contaminated or emergency surgery remains insufficient.

**Systematic review registration:**

PROSPERO CRD42025632413.

**Supplementary Information:**

The online version contains supplementary material available at 10.1007/s10029-025-03533-2.

## Background

Incisional hernias (IH) are a frequent complication following laparotomy, occurring in 5–30% of patients and leading to significant postoperative disability and costs [1, 2]. Indeed, IH result in chronic pain, dysfunction, the need for reoperation, and prolonged hospital stays, ultimately increasing healthcare costs [3]. The prevention of IH has therefore become a major focus in surgical practice, particularly for high-risk patients who undergo laparotomy. Current clinical guidelines from the European and American Hernia Societies recommend prophylactic mesh reinforcement (PMR) in laparotomy closures for patients at high risk of hernia development, such as those with obesity, obstructive pulmonary disease, diabetes, or previous abdominal surgeries [4]. However, despite its widespread use in elective surgery, clear guidelines and recommendations for the use of mesh reinforcement in emergency laparotomies are lacking. Moreover, emergency laparotomy patients often present with a contaminated field and major comorbidities, that make them more vulnerable to adverse outcomes, including mesh complications, and contribute to significant postoperative morbidity [5, 6].

Among the various techniques for PMR, the retromuscular or sublay technique is regarded as one of the most effective [7, 8]. This approach involves placing the mesh behind the abdominal wall musculature and above the posterior fascia, providing reinforcement to the incision closure while avoiding direct contact with the viscera. The retromuscular placement of prophylactic mesh offers several recognized advantages. These include strong biomechanical reinforcement of the abdominal wall and lower rates of mesh-related complications such as adhesions, bowel obstruction, and fistula formation compared to intraperitoneal positions. Furthermore, the retromuscular plane is considered well-vascularized, which may reduce the risk of infection compared to onlay techniques. Reduced rates of IH and fewer complications were reported when compared to other mesh techniques, such as the onlay or intraperitoneal placements. However, the evidence supporting the widespread use of retromuscular PMR remains fragmented, with studies providing mixed results regarding its effectiveness and safety. The procedure is more technically demanding and typically associated with longer operative times. Placement of mesh, even in this 'protected' plane, carries a risk of postoperative complications such as seroma, hematoma, infection, and, albeit rarely, mesh explanations. In contaminated or emergency settings, the risk of infection and complications may be increased, and the long-term benefit remains less well established. Moreover, once the retromuscular plane has been opened and used for prophylactic mesh placement, this anatomical space is no longer available for future repairs in the event of recurrence or other abdominal wall problems, potentially limiting options for secondary surgical interventions.

Thus, while retromuscular PMR shows promise as a strategy for preventing IH, there is a need for a comprehensive evaluation of its impact on both elective and contaminated/emergency laparotomy patients. A systematic review and meta-analysis are warranted to synthesize existing evidence and provide a clearer understanding of the role of retromuscular PMR in reducing IH rates and improving surgical outcomes. This work will also help clarify any potential risks, such as surgical site infection (SSI), seromas, and hematomas compared to primary suture closure (PSC) in laparotomy procedures.

## Study aim

The aim of this systematic review and meta-analysis was to evaluate the effectiveness and safety of retromuscular PMR in preventing IH following both elective and contaminated/emergency laparotomy procedures. This study compared the incidence of IH and examined secondary outcomes such as SSI, seromas, and hematomas between retromuscular PMR and PSC.

## Materials and methods

This systematic review adhered to the Preferred Reporting Items for Systematic Review and Meta-Analysis (PRISMA 2020) guidelines [9] and complied with the Meta-Analysis of Observational Studies in Epidemiology (MOOSE) Checklist [10] (Supplementary material 1) and the Assessing the Methodological Quality of Systematic Reviews (AMSTAR) guidelines [11] (Supplementary material 2).

Relevant studies were identified through comprehensive searches in electronic databases, including MEDLINE, Embase, Web of science and Cochrane Library. Reference lists from previous reviews and all included studies were searched for any additional studies. Corresponding authors were contacted when necessary to obtain additional information or clarify study eligibility. The last date of search was March 7th, 2025. The detailed search strategy included a combination of the Medical subject Headungs (MeSH terms), Emtree terms, and free text. It is reported as Supplementary material 3. The study was registered at the International Prospective Register of Systematic Reviews (PROSPERO: CRD42025632413) prior to its initiation.

### Eligibility criteria

The search followed the following PICO framework:

- **Population (P):** Patients undergoing elective or emergency midline laparotomy.
- **Intervention (I):** Retromuscular prophylactic mesh reinforcement.
- **Comparison (C):** Primary suture closure without mesh reinforcement.
- **Outcome (O):** Incisional hernia rates, with secondary outcomes including SSI, seromas, and hematomas.

Inclusion criteria: Only randomized controlled trials (RCTs) written in English were retained for the meta-analysis to ensure the highest level of evidence and minimize the risk of bias inherent in observational studies. Studies were eligible for inclusion if they enrolled adult patients undergoing elective or emergency midline laparotomy for any abdominal surgical indication and implemented PMR in the retromuscular (sublay) position during fascial closure. Eligible studies were required to compare PMR with PSC without mesh reinforcement. To be included, studies had to report the incidence of incisional hernia at any follow-up interval. Secondary outcomes of interest included the occurrence of SSI, seroma, and hematoma formation. When two or more publications reported data from the same study population, both were included in the review, provided they presented distinct follow-up periods. For example, if one article reported the initial results and a subsequent publication presented the long-term follow-up of the same cohort, the first was used to extract short- or mid-term outcomes, while the second was used to extract long-term outcomes.

Exclusion criteria: Studies were excluded if they were not published in English or consisted solely of study protocols, editorials, letters, conference abstracts, systematic reviews, or meta-analyses. Additionally, studies were excluded if prophylactic mesh was placed in a position other than the retromuscular (sublay) plane—such as onlay, intraperitoneal, or inlay techniques—or if they lacked a comparator group treated with PSC alone.

### Data extraction

Data extraction was independently performed by two reviewers (ML and RS, a surgery resident and an attending physician) using Covidence [12], a web-based platform designed to streamline systematic and literature reviews. Reviewers were blinded to study authors and journal titles. Any discrepancies were resolved through consensus.

The following variables were extracted: study authors, year of publication, country, study design, sample size, patient demographics (sex, age, body mass index [BMI]), surgical indication, mesh type (synthetic or biologic), PSC technique, and postoperative outcomes. These variables were selected based on their relevance to evaluating the effectiveness and safety of PMR, and in alignment with prior systematic reviews on the topic. Where available, outcome data were extracted at standardized follow-up intervals (6, 12, 24, and 60 months) to facilitate consistent comparisons across studies. In studies reporting outcomes for multiple mesh placement techniques (e.g., onlay, intraperitoneal, retromuscular), only data specific to the retromuscular (sublay) group were extracted and included in the analysis.

### Outcomes

The primary outcome was the incidence of incisional hernia, identified through clinical examination or imaging (ultrasound or CT scan) at any available follow-up, directly addressing the clinical hypothesis. Secondary outcomes included SSI, seroma, and hematoma formation, chosen for their importance in assessing the safety profile of PMR.

### Quality assessment

Two authors (ML and RS) independently assessed the methodological quality of the included RCT using the Cochrane Risk of Bias 2.0 tool [13]. This validated tool evaluated multiple domains of potential bias, including randomisation process, deviation from the intended intervention, missing outcome data, measurement of the outcome and selection of the reported result. Based on these assessments, each study was assigned an overall risk of bias category: low, moderate, or high. Any disagreements among reviewers were resolved through discussion.

### Statistical analysis

Statistical analysis was performed using the Mantel–Haenszel random-effects model to estimate pooled odds ratios (ORs) with 95% confidence intervals (CIs). The random-effects model, based on the DerSimonian and Laird method, was selected to account for expected clinical and methodological heterogeneity among studies. Subgroup analyses were performed to distinguish between indications, mesh type, BMI, and contamination. Elevated BMI was defined as > 30 kg/m^2^. For subgroup analyses, each study was assigned to the surgical indication that represented the majority of its included population. Specifically, if more than 50% of the patients in a study underwent laparotomy for a particular indication (e.g., contaminated/emergency field), the study was classified and analysed within that subgroup. Emergency laparotomy and contaminated field were grouped into a single category, as they represent similar operative challenges in terms of tissue quality, infection risk, and surgical complexity.

Heterogeneity was assessed using Cochran’s Q (Chi^2^) test and quantified with the I^2^ statistic, where an I^2^ value greater than 50% was considered to indicate substantial heterogeneity. Between-subgroup differences were evaluated with a Chi^2^ test for subgroup interaction. To explore the presence of potential publication bias or small-study effects, a funnel plot was visually inspected for asymmetry. In addition, Egger’s regression test was performed to statistically assess funnel plot asymmetry. All analyses were performed using Review Manager (RevMan) [14] and R software program (version 4.5.1) [15], and statistical significance was defined as a two-sided P-value less than 0.05.

## Results

### Systematic review

**Fig. 1:**
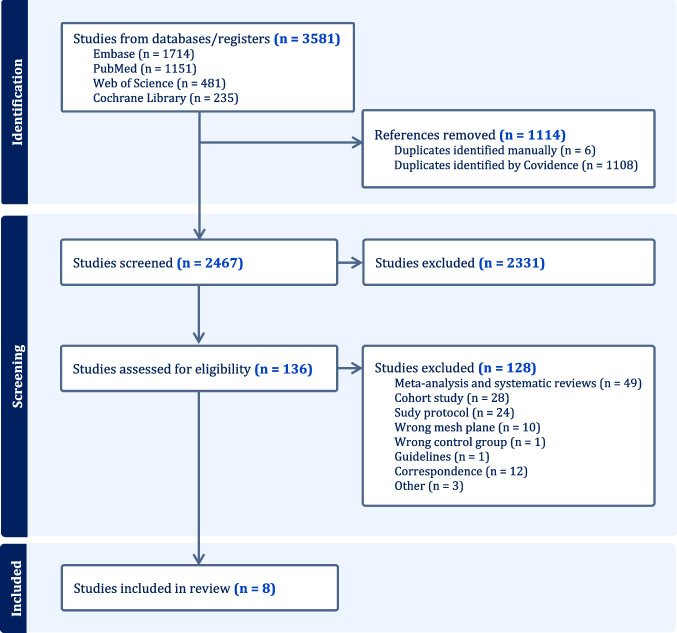
Prisma diagram

A comprehensive literature search was performed using four electronic databases: Embase (*n* = 1714), PubMed (*n* = 1151), Web of science (*n* = 481), and the Cochrane Library (*n* = 235) for a total of 3581 records. After the removal of 1114 duplicates (1108 identified by Covidence and 6 manually), 2467 studies remained for screening. Title and abstract screening further excluded 2331 records, and 136 full-text articles were assessed for eligibility. Among these, 128 were excluded for reasons including wrong study type (meta-analyses, protocols, correspondence, or guidelines), wrong intervention (e.g., mesh in a plane other than retrorectus), or inappropriate comparator groups. Ultimately, 8 studies met all inclusion criteria and were included in the systematic review: Strzelczyk et al. [16], Sarr et al. [17], Muysoms et al. [18], Dewulf et al. [19], Jairam et al. [20], Van den Dop et al. [21]. Pizza et al. [22], Coelho et al. [23]. The PRISMA flow chart is reported in Fig. 1.

The studies were conducted across Europe [16, 18–22] and North America [17, 23], involving patients undergoing midline laparotomy for various indications, including abdominal aortic aneurysm (AAA) [18–21], bariatric surgery [16, 17], high-risk patients (overweight/obese, current smoker) [23], and clean-contaminated emergency procedures [22, 23]. They were published between 2006 and 2023 and enrolled a total of 1167 patients (614 mesh closure, 553 primary closure). Dewulf et al. reported long term follow-up from the PRIMAAT trial from Muysoms et al., while Van den Dop et al. reported long term follow-up from the PRIMA trial from Jairam et al.

Most studies employed a polypropylene mesh. A polyester mesh was used by Pizza et al., Sarr et al. used a biologic mesh (Surgisis Gold), while Coelho et al. evaluated both biologic mesh (Porcine acellular matrix) and small vs. large bite closure techniques. Table 1 presents the baseline characteristics of the included studies, detailing country of origin, surgical indications, and technical aspects such as mesh type and overlap, placement technique, and closure method. The duration of postoperative follow-up ranged from 6 to 60 months.

**Table 1:** Baseline characteristics of the included studies

Author	Year	Country	Indication of surgery	*N* patients (sublay mesh)	*N* patients (Suture)	Mesh type	Mesh fixation	Posterior sheet closure	Anterior sheet closure	Mesh overlap	Primary suture technique
Strzelczyk [16]	2006	Poland	Open Roux-en-Y gastric bypass	36	38	Polypropylene (not specified)	PDS	PDS	Vicryl	4 cm	Running Vicryl
Sarr [17]	2014	USA	Open Roux-en-Y gastric bypass	185	195	Biologic (Surgisis Gold)	Sutures (not specified)	Not specified	Not specified	4 cm	Running Nylon/Vicryl/PDS
Muysoms [18] and Dewulf [19]	2016 and 2022	Belgium, Netherlands	Elective AAA repair	56	58	Polypropylene (Ultrapro)	Not specified	PDS	PDS	3.75 cm	Running PDS
Jairam [20] and Van den Dop [21]	2017 and 2024	Netherlands, Germany, Austria	Elective laparotomy (AAA/BMI ≥ 27)	185	107	Polypropylene (Optilene)	Fibrin sealant (Tisseel)	MonoPlus	MonoPlus	3 cm	Running MonoPlus
Pizza [22]	2021	Italy	Urgent clean-contaminated laparotomy	100	100	Polyester (Parietex ProGrip)	Self-fixating	PDS	PDS	2 cm	Double layer running PDS
Coelho [23]	2023	USA	Overweight patients (BMI 25 kg/m or greater), current smoker, or undergoing a contaminated case (wound class II to IV) undergoing LT	52	55	Biologic (Porcine acellular dermal matrix)	PDS	Vicryl	PDS	3 cm	Small bites 2–0 PDS vs large bites 0 PDS

The included studies featured two distinct patient populations: a younger cohort with elevated BMI, primarily undergoing bariatric or general abdominal surgery, and an older cohort undergoing AAA repair. The younger population included patients from the studies by Strzelczyk et al., Sarr et al., and Coelho et al. These patients had a mean age ranging from 39.1 to 50.5 years, and were characterized by markedly elevated BMIs (mean values between 32 and 48.2 kg/m^2^). Male sex predominance was present in most cases, notably 63.5% in the Strzelczyk et al. study. Comorbidity data were limited in this group, though smoking, diabetes and immunosuppression were generally not reported. In contrast, the older patient group was represented by the studies of Muysoms et al. and Dewulf et al., Jairam et al. and Van den Dop et al., and Pizza et al., where the mean age ranged from 64.7 to 72 years. These patients had lower average BMIs (25.5 to 30.4 kg/m^2^) and higher rates of reported chronic comorbidities. Notably, in the Muysoms et al./Dewulf et al. cohort, 65% were current smokers, and 31% had chronic obstructive pulmonary disease (COPD). Similarly, the Pizza et al. and Jairam et al./Van den Dop et al. studies reported substantial proportions of COPD (10–31%), diabetes (12–20%), and a small percentage of immunosuppression. Prior midline incisions were reported in 2% and 11.5% of patients in the Muysoms et al./Dewulf et al. and Pizza et al. cohorts, respectively. This clear demographic and clinical dichotomy underscores the heterogeneity of populations undergoing PMR, which may influence the risk profile and surgical outcomes across studies. Table 2 provides an overview of the baseline characteristics of the included population. Across the six studies, follow-up modalities varied consistently and included clinical examination by a surgeon, ultrasound, CT, and telephone contact with primary care providers. Table 3 summarizes follow-up methods and periods.

**Table 2:** Demographical characteristics of the included population expressed as mean ± SD, median (range) and number (%)

Author	Mean age	Male	BMI	Current smoker	COPD	Diabetes	Immunosuppression	Previous midline incision
Strzelczyk [16]	39.1 ± 12.0	47 (63.5)	46.5 ± 7.4					
Sarr [17]	44.9 ± 11.4	41 (10.8)	48.2 ± 8.0					
Muysoms [18] and Dewulf [19]	72 ± 8.0	105 (92)	25.5 ± 3.6	69 (65)	34 (31)	19 (17)	0 (0)	2 (2)
Jairam [20] and Van den Dop [21]	64.7 ± 10.5	176 (60)	30.4 ± 4.9	61 (21)	28 (10)	58 (20)		11 (4)
Pizza [22]	66.0 (range 19–88)	86 (43)	28.7 (range 19–34)	29 (14.5)	21 (10.5)	24 (12)	5 (2.5)	23 (11.5)
Coelho [23]	50.5 ± 13.5	48 (45)	32 ± 7.5	17 (16)

**Table 3:** Follow-up methods and period. US = ultrasound, S = clinical examination performed by surgeon, P = phone call, GP = General Practitioner, CT = CT scan

Author	Follow-up modality	6 months follow-up	12 months follow-up	24 months follow-up	60 months follow-up
Strzelczyk [16]	US, S			x	
Sarr [17]	S, P, GP	x	x	x	
Muysoms [18]	S		x	x	
Dewulf [19]	S				x
Jairam [20]	US, CT, S			x	
Van den Dop [21]	US, CT, S				x
Pizza [22]	US, S	x	x	x	
Coelho [23]	US, S	x			

### Meta analysis

The primary outcome—the incidence of IH—was analysed according to different follow-up durations (6, 12, 24, and 60 months, as well as longest reported follow-up). Sub-group analysis was performed according to procedure setting (contaminated/emergency vs. elective) and mesh type (biologic vs. synthetic).

#### Six months follow-up, Fig. 2

**Fig. 2:**
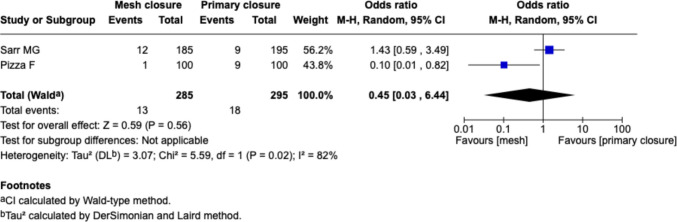
Forest plot for the comparison of incisional hernia rate in PMR vs. primary closure at 6 months follow-up

Two studies (Sarr et al. and Pizza et al.) reported IH rates at 6 months. The pooled odds ratio (OR) for IH with mesh closure versus PSC was 0.45 [95% CI: 0.03–6.44], with no statistically significant difference (*p* = 0.56). Heterogeneity was high (I^2^ = 82%).

#### Twelve months follow-up, Fig. 3

**Fig. 3:**
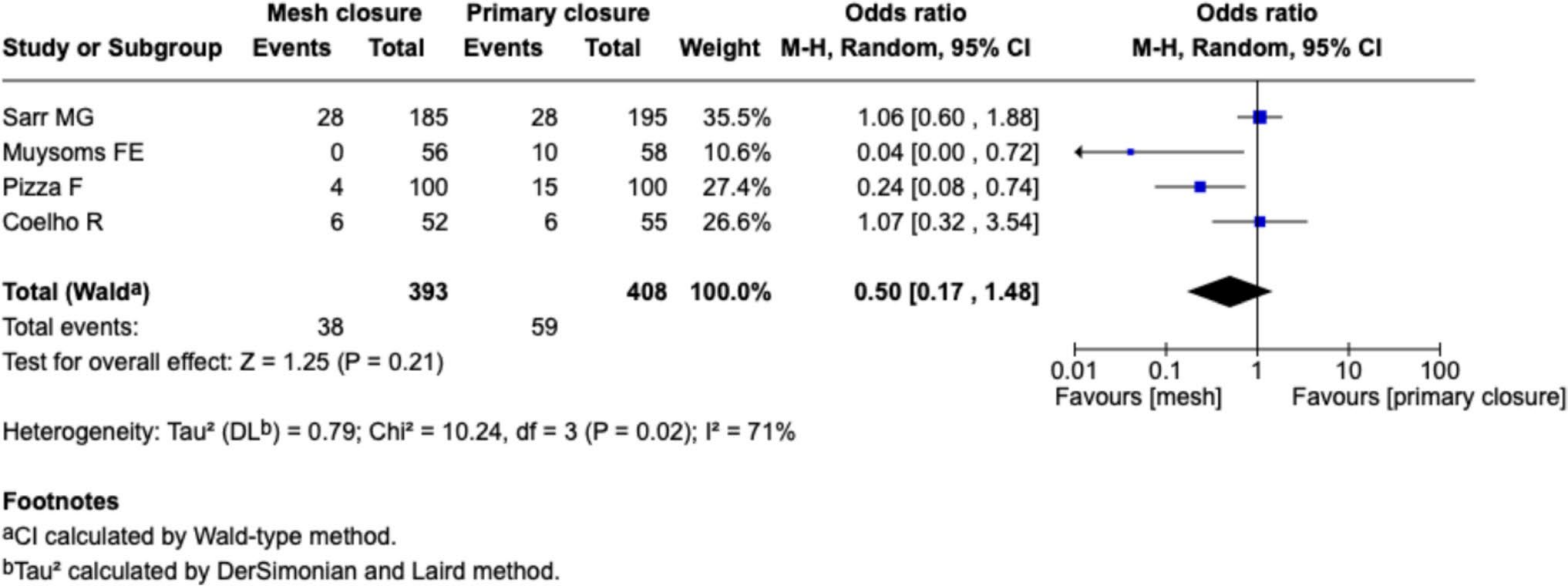
Forest plot for the comparison of incisional hernia rate in PMR vs. primary closure at 12 months follow-up

Four studies reported outcomes at 12 months (Sarr et al., Muysoms et al., Pizza et al., Coelho et al.). The pooled estimate showed a non-significant reduction in IH with mesh closure (OR 0.50 [0.17–1.49], *p* = 0.21). Heterogeneity was high (I^2^ = 71%). While Muysoms et al. reported no hernias in the mesh group, Coelho et al. observed similar event rates between groups.

#### Twenty-four months follow-up, Fig. 4

**Fig. 4:**
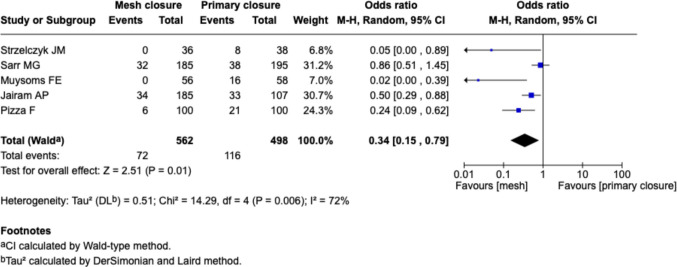
Forest plot for the comparison of incisional hernia rate in PMR vs. primary closure at 24 months follow-up

Five studies reported outcomes at or near 24 months (Strzelczyk et al., Sarr et al., Muysoms et al., Jairam et al. and Pizza et al.). The meta-analysis revealed a significant reduction in IH in the mesh group compared to suture closure (OR 0.34 [0.15–0.79], *p* = 0.01). The effect was consistent across studies, with high heterogeneity (I^2^ = 72%). Notably, two studies (Muysoms et al. and Strzelczyk et al.) reported extreme results with no hernia events in the mesh group.

#### Sixty months follow-up, Fig. 5

**Fig. 5:**
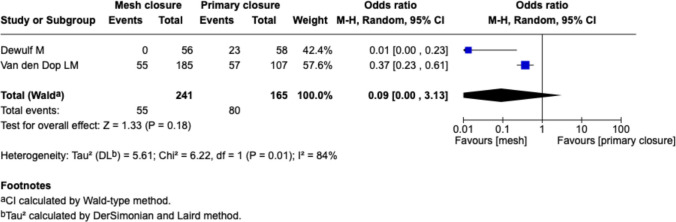
Forest plot for the comparison of incisional hernia rate in PMR vs. primary closure at 60 months follow-up

Only two studies (Dewulf et al. and Van den Dop et al.) reported long-term outcomes at 5 years. The pooled estimate showed a trend toward fewer IH with mesh (OR 0.09 [0.00–3.13], *p* = 0.18), though this was not statistically significant. Heterogeneity was high (I^2^ = 84%).

#### Longest reported follow-Up, Fig. 6

**Fig. 6:**
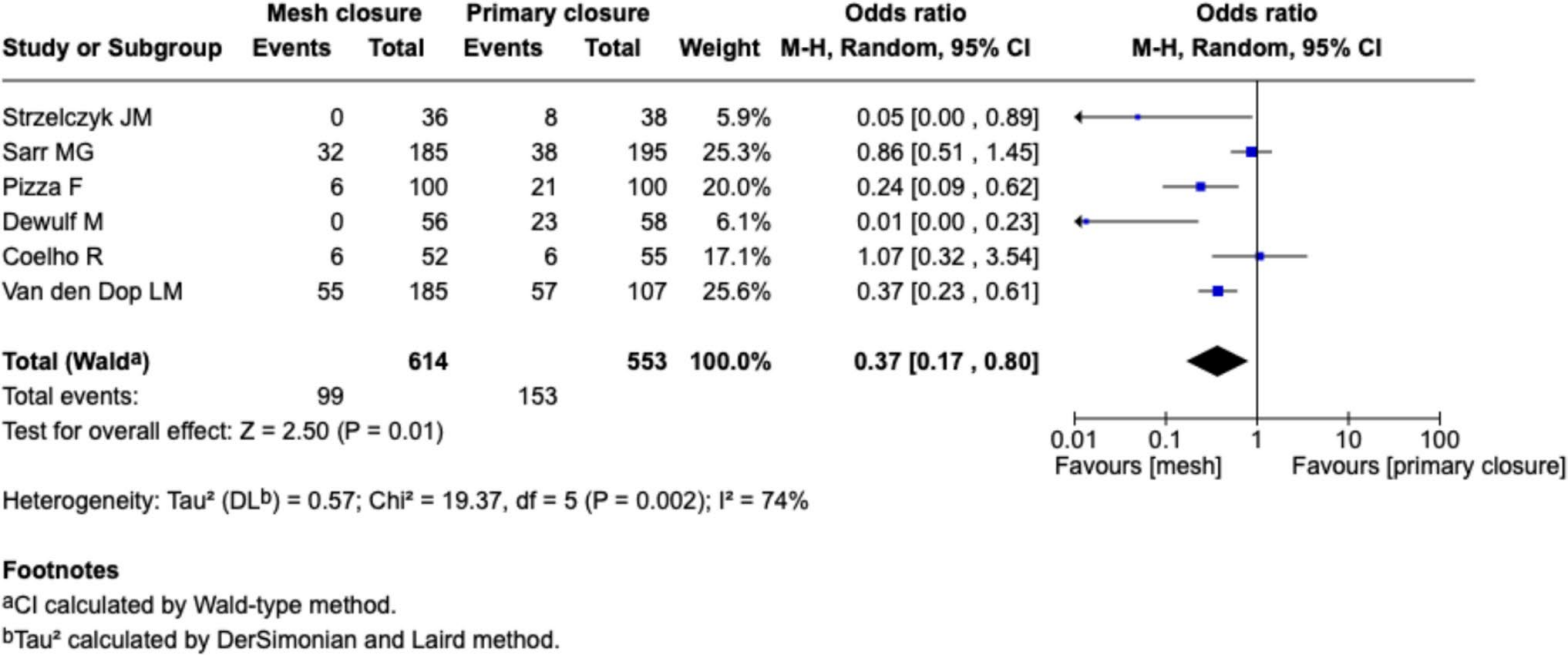
Forest plots for the comparison of incisional hernia rate in PMR vs. primary closure at longest reported follow-up with risk of bias

In an aggregated analysis using the longest available follow-up for each study, mesh reinforcement significantly reduced the risk of IH compared to primary closure (OR 0.37 [0.17–0.80], *p* = 0.01). Heterogeneity was high (I^2^ = 74%).

#### Sub-groups analysis: surgical indication, Fig. 7

**Fig. 7:**
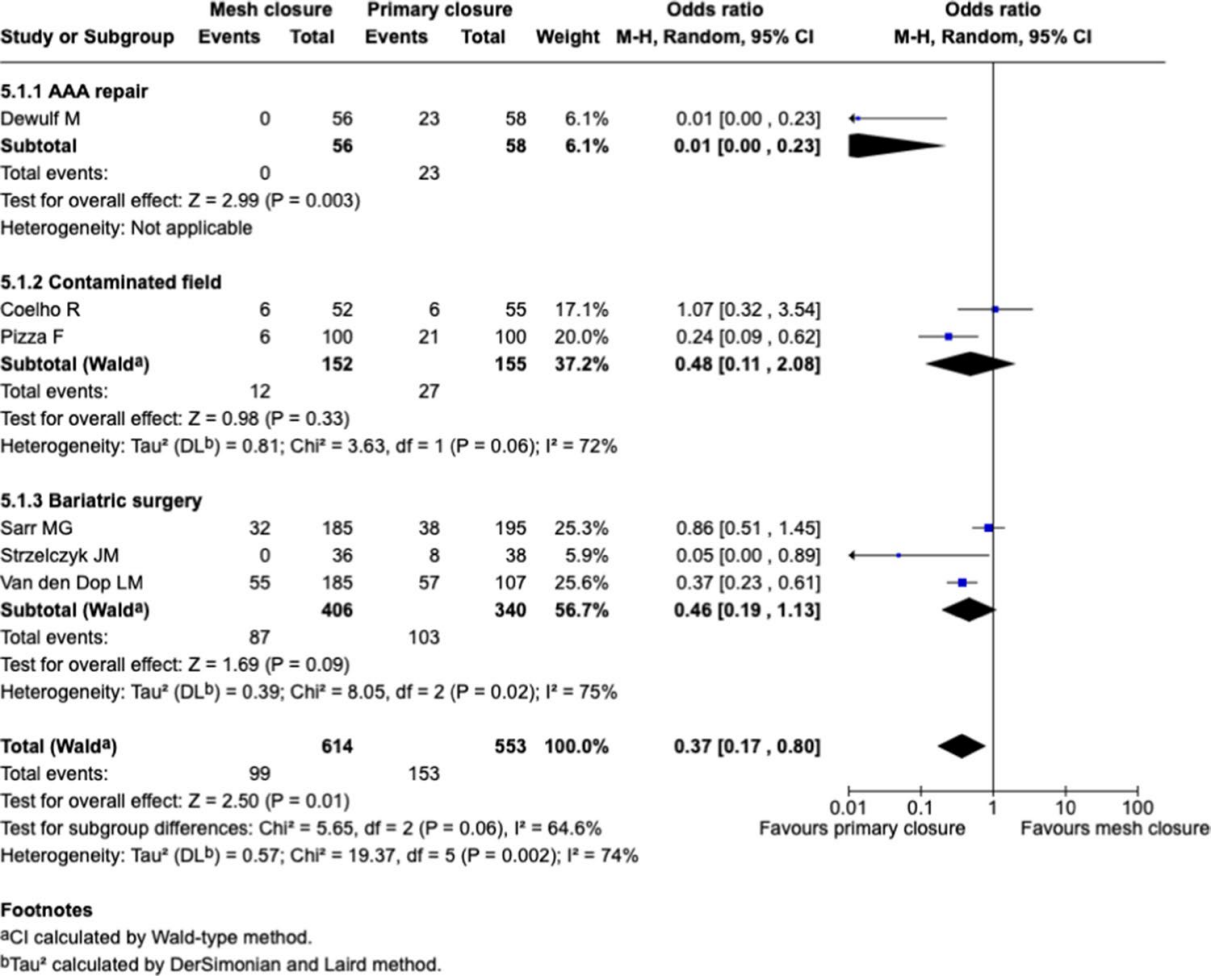
Forest plots for the comparison of incisional hernia rate in PMR vs. primary closure at longest reported follow-up with sub-groups analysis for indication

Studies were stratified according to surgical indication, including AAA repair, bariatric surgery, and contaminated surgical fields. A significant benefit of PMR was observed in the including AAA repair subgroup (OR 0.01 [95% CI: 0.00–0.23], *p* = 0.003). However, it is important to note that only the study by Dewulf et al. reported data specific to AAA repair, limiting the generalizability of this finding. Subgroup heterogeneity was substantial, with an I^2^ value of 64%, indicating considerable variability between subgroups.

#### Sub-groups analysis: mesh type, Fig. 8

**Fig. 8:**
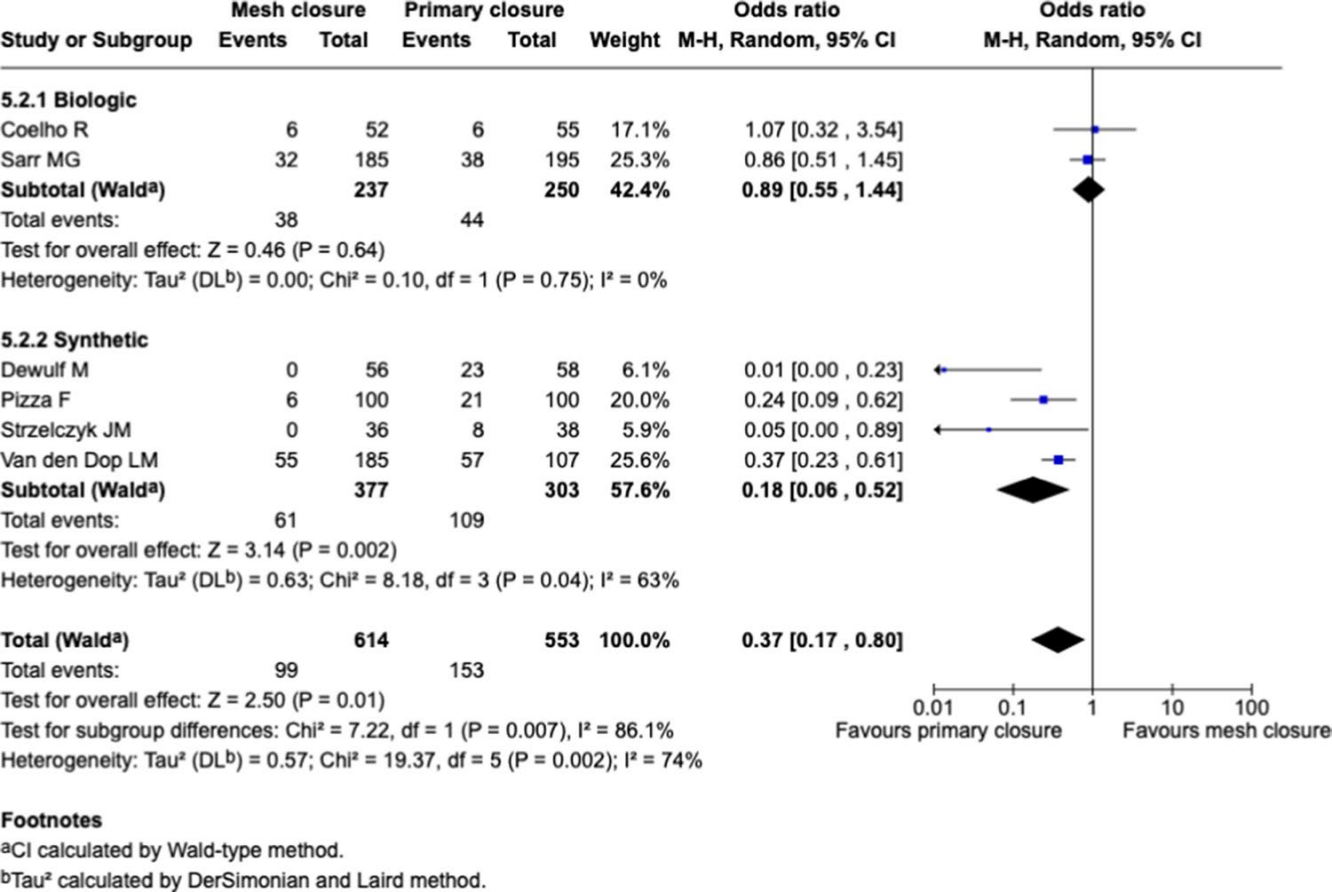
Forest plots for the comparison of incisional hernia rate in PMR vs. primary closure at longest reported follow-up with sub-groups analysis for mesh type

Subgroup analysis based on mesh type revealed a significant benefit associated with synthetic mesh use (OR 0.18 [95% CI: 0.06–0.52], *p* = 0.002). In contrast, no significant difference was observed with biologic mesh (OR 0.89 [95% CI: 0.55–1.44], *p* = 0.64). A statistically significant difference between subgroups was detected (I^2^ = 86.1%, *p* = 0.007), indicating substantial heterogeneity between synthetic and biologic mesh outcomes. Heterogeneity within subgroups was moderate for synthetic mesh (I^2^ = 63%) and absent for biologic mesh (I^2^ = 0%).

#### Sub-groups analysis: BMI, Fig. 9

**Fig. 9:**
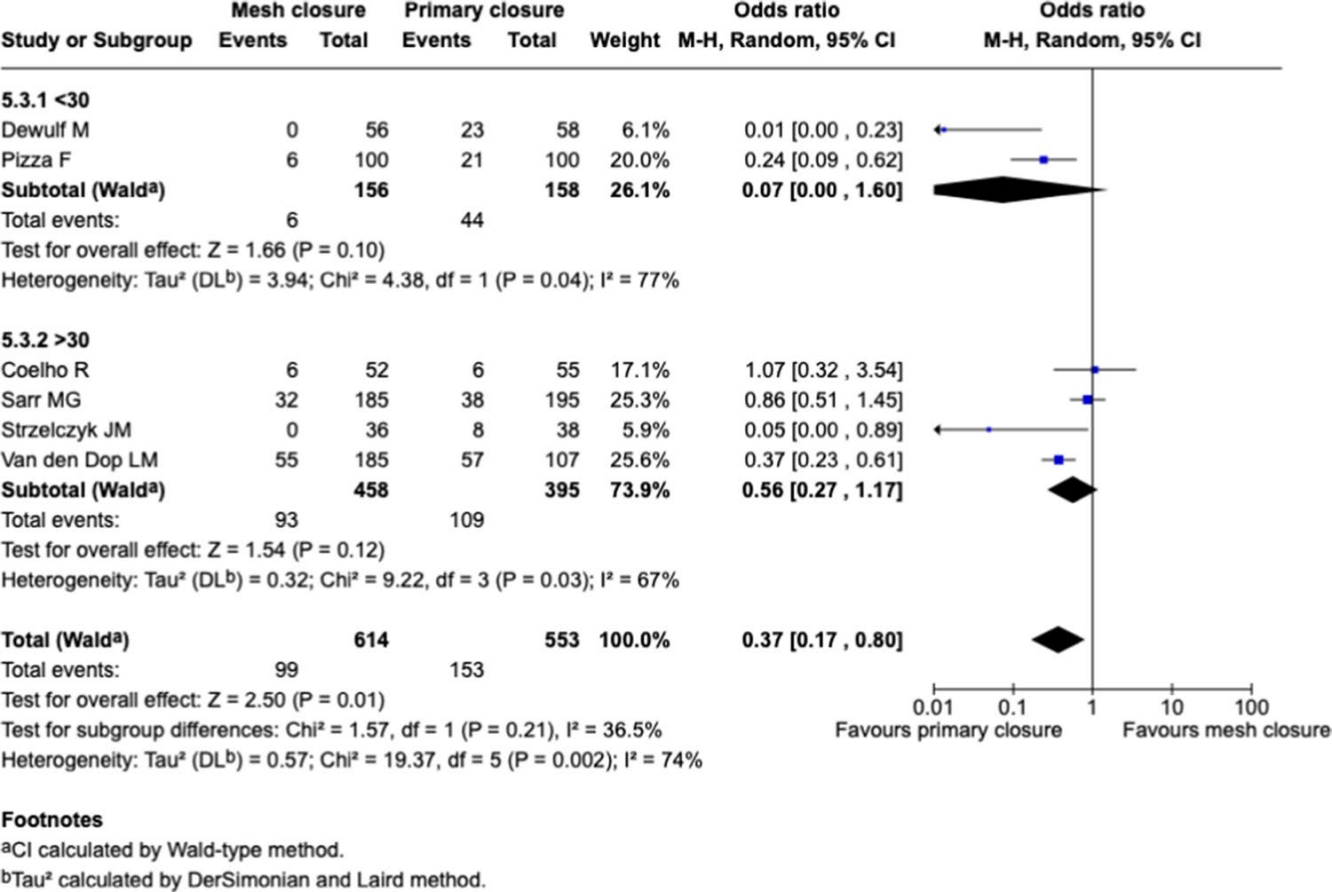
Forest plots for the comparison of incisional hernia rate in PMR vs. primary closure at longest reported follow-up with sub-groups analysis for BMI

The benefit was absent in both elevated (OR 0.56 [0.27–1.17], *p* = 0.12; I^2^ = 67%) and normal BMI (OR 0.07 [0.00–1.60], *p* = 0.1; I^2^ = 77%), with low subgroup differences (I^2^ = 36.5%, *p* = 0.21).

#### Sub-groups analysis: emergency and contaminated field, Fig. 10

**Fig. 10:**
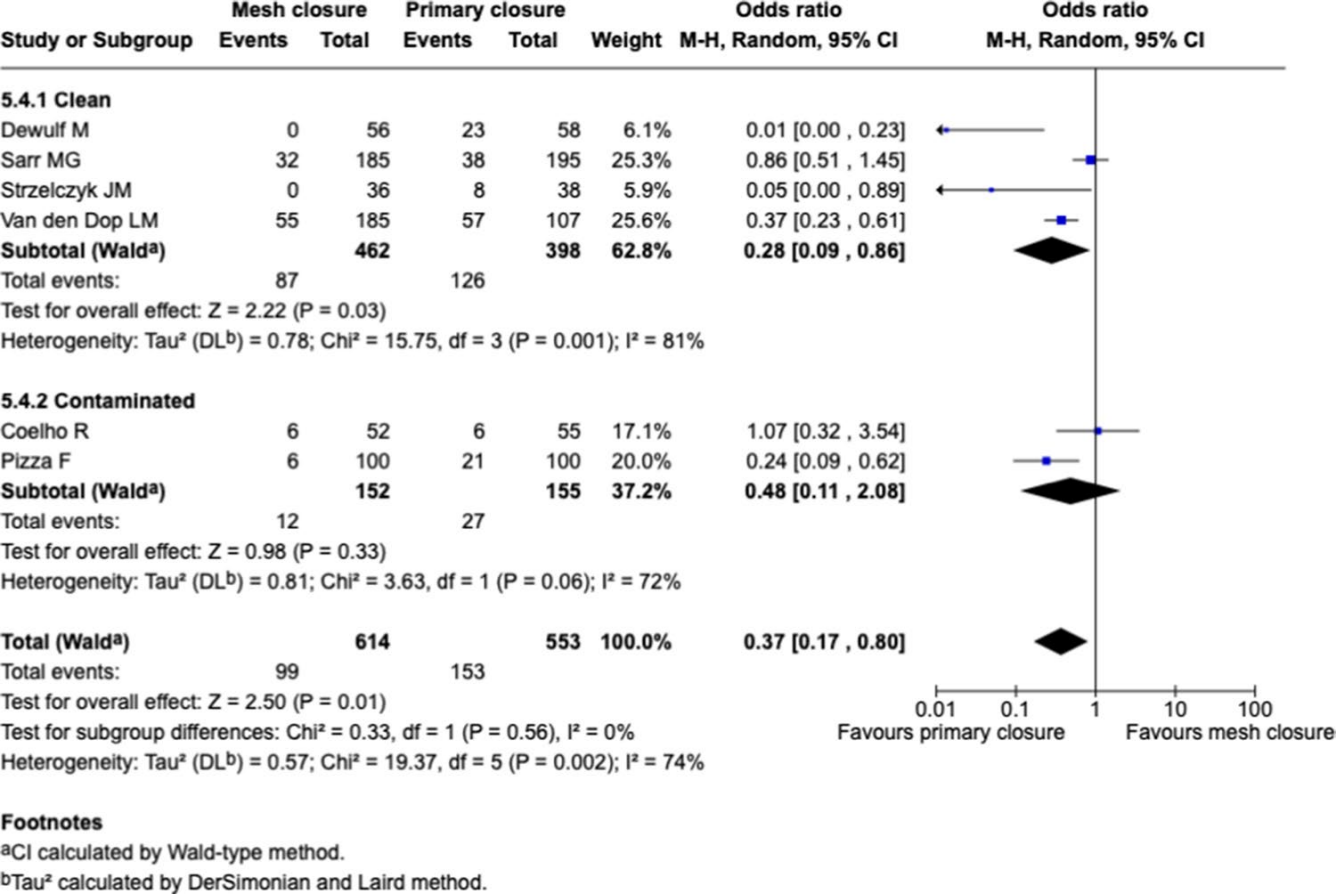
Forest plots for the comparison of incisional hernia rate in PMR vs. primary closure at longest reported follow-up with sub-groups analysis for contamination level

The benefit was significant when restricted to clean field (OR 0.28 [0.09–0.86], *p* = 0.03; I^2^ = 81%), while no significant difference was observed with contaminated/emergency field (OR 0.48 [0.11–2.08]; *p* = 0.33, I^2^ = 72%). There was no statistically significant interaction between subgroups (Chi^2^ = 0.33, *p* = 0.56), suggesting no clear evidence that the effect of mesh differs by contamination status.

#### Secondary outcomes

##### Surgical site infection, Fig. 11

**Fig. 11:**
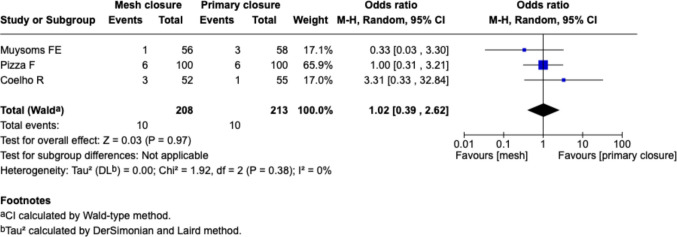
Forest plot for the comparison of surgical-site infection rate in PMR vs. primary closure

Three studies (Muysoms et al., Pizza et al., Coelho et al.) reported SSI rates. There was no significant difference in SSI between mesh and suture groups (OR 1.02 [0.39–2.62], *p* = 0.97). Heterogeneity was negligible (I^2^ = 0%), and event rates were low across all studies.

##### Seroma, Fig. 12

**Fig. 12:**
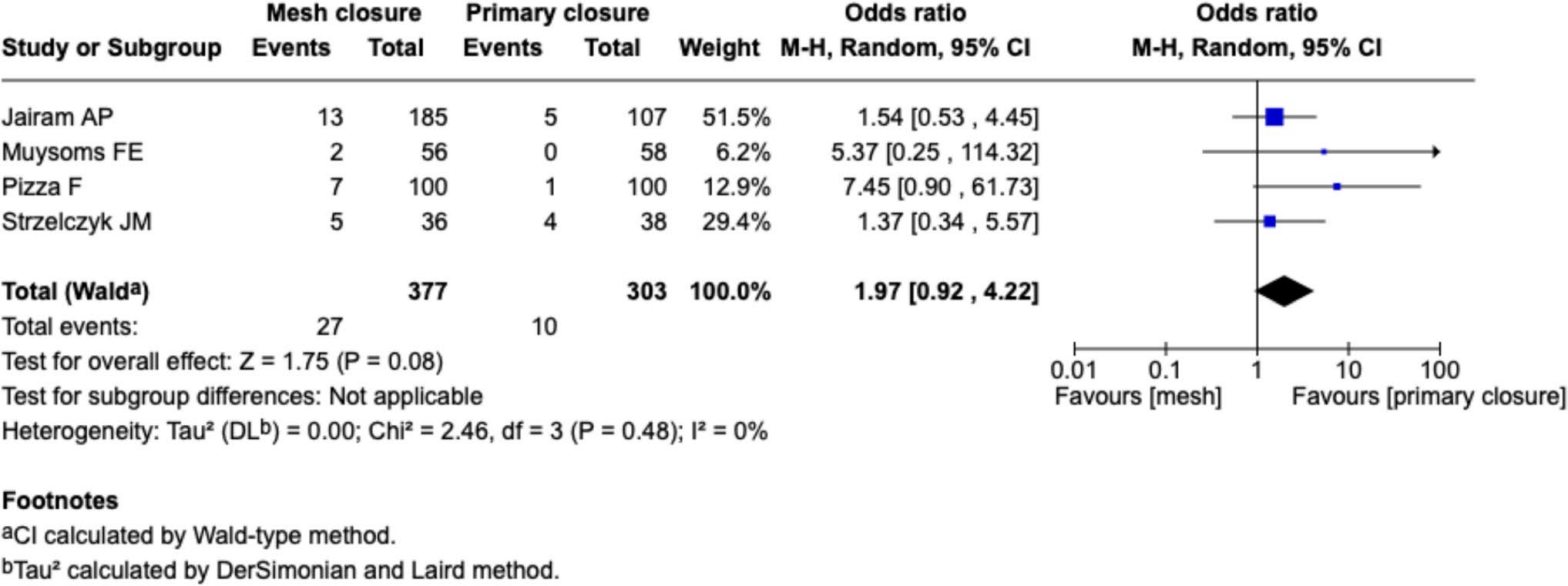
Forest plot for the comparison of seroma rate in PMR vs. primary closure

Four studies (Jairam et al., Muysoms et al., Pizza et al., Strzelczyk et al.) reported seroma rates. The pooled odds ratio was 1.97 [0.92–4.22] (*p* = 0.08), indicating a non-significant trend toward increased seroma formation in the mesh group. Heterogeneity was low (I^2^ = 0%), and confidence intervals were wide due to sparse events.

##### Hematoma, Fig. 13

**Fig. 13:**
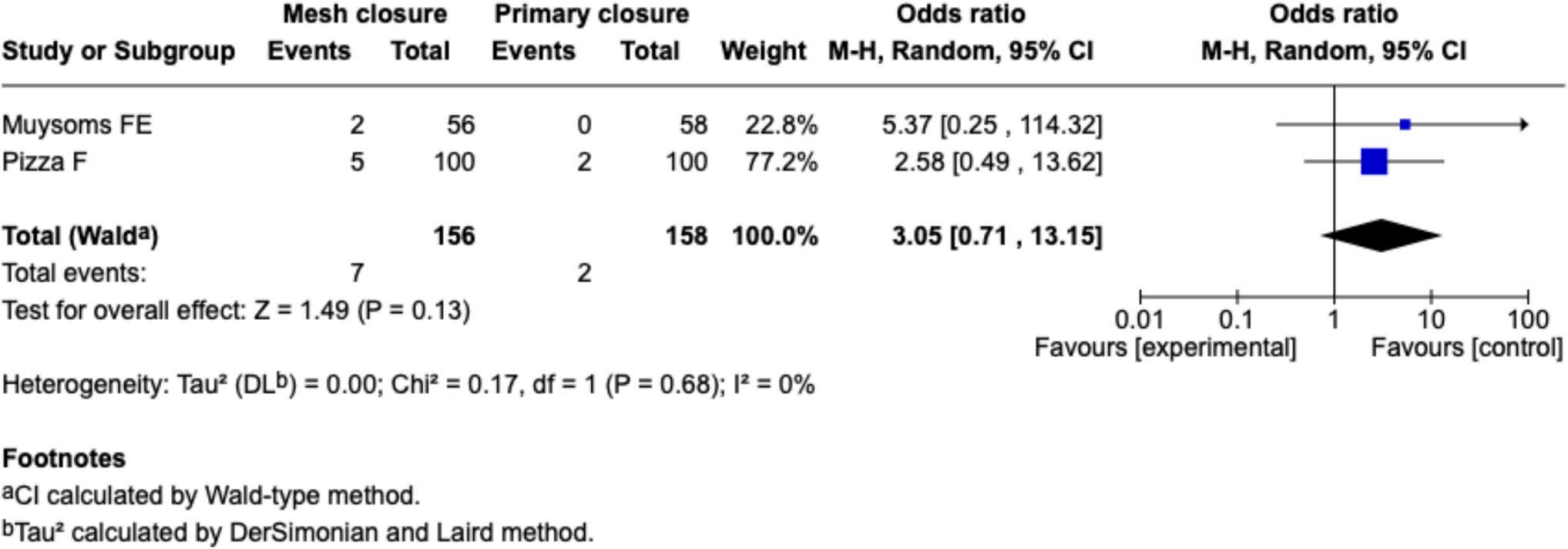
Forest plot for the comparison of hematoma rate in PMR vs. primary closure

Only two studies (Muysoms et al., Pizza et al.) reported hematoma formation. The pooled estimate showed no significant difference (OR 3.05 [0.71–13.15], *p* = 0.13). Despite a numerical trend toward more hematomas in the mesh group, confidence intervals were wide and not statistically meaningful.

#### Other outcomes

Muysoms et al. reported a significantly longer operative time in the mesh group (mean difference 16 min). Only a few studies documented reoperations: Sarr et al. reported that 5 reoperations occurred in the suture group versus none in the mesh group, Pizza et al. reported one reoperation due to deep mesh infection and one mesh removal.

### Risk of bias

**Fig. 14:**
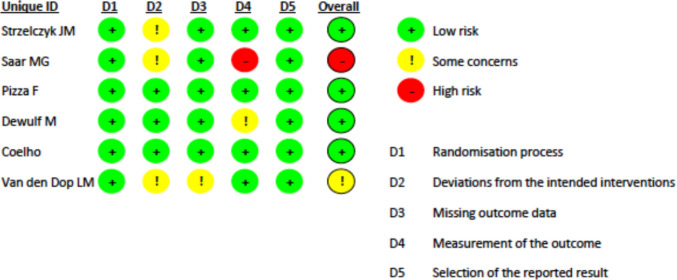
Risk of Bias 2 Tool

Risk of bias (Fig. 14) was evaluated across standard methodological domains, including randomisation process, deviation from the intended intervention, missing outcome data, measurement of the outcome and selection of the reported result. Most studies were judged to have a low risk of bias in randomization and outcome reporting. Due to the nature of the intervention, blinding of participants and outcome assessors was generally not feasible, contributing to performance and detection bias in several studies. Attrition bias was considered low in most trials, supported by adequate follow-up and the use of intention-to-treat analyses.

### Publication bias

**Fig. 15:**
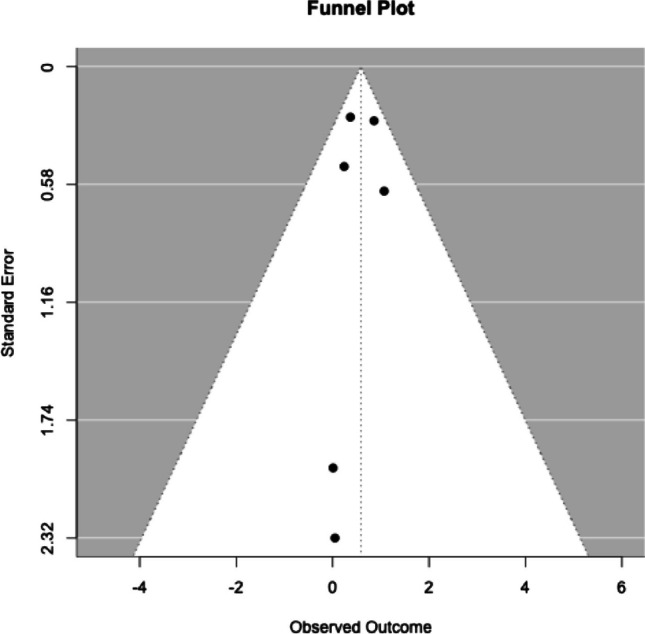
Funnel plot constructed using the longest available follow-up for each study

Visual inspection of the funnel plot, constructed using the longest available follow-up from each study, revealed some asymmetry in the distribution of studies around the overall effect estimate (Fig. 15). Specifically, two large studies were located to the right of the pooled effect, while two large and two small studies were positioned to the left, suggesting an imbalance in the spread of effect sizes. Although this visual asymmetry could raise concerns about potential small-study effects or publication bias, Egger’s regression test for funnel plot asymmetry was not statistically significant (t = -0.22, *p* = 0.83). Therefore, the presence of a publication bias effect cannot be confirmed.

## Discussion

This meta-analysis demonstrates that retromuscular PMR significantly reduces the incidence of IH following midline laparotomy, with the most consistent benefit observed at longer follow-up intervals. However, the interpretation of these findings is tempered by substantial heterogeneity across studies (I^2^ ≥ 70%), which limits the certainty and generalizability of the pooled estimates. Several clinical and methodological factors likely contribute to this variability.

### Patient population

The included studies encompassed two distinct patient cohorts: younger individuals with elevated BMI undergoing bariatric or general abdominal surgery, and older patients with comorbidities undergoing AAA repair. These cohorts had different high-risk profiles. Indeed, obesity and increased intra-abdominal pressure predispose bariatric patients to fascial dehiscence and hernia formation, while AAA patients often exhibit compromised connective tissue integrity and higher rates of smoking and COPD, which impair fascia and wound healing. These baseline differences likely influence the effectiveness of PMR and contribute to inter-study heterogeneity.

### Surgical technique

Variations in operative technique further complicate comparisons. Although all studies employed retromuscular mesh placement, differences in mesh fixation (e.g., sutures vs. self-gripping), overlap, fascial closure methods (e.g., small vs. large bites), and surgeon experience may affect outcomes. Some trials reported standardized closure protocols, while others lacked detailed descriptions. Such operator-dependent factors are known to influence recurrence and complication rates and likely contribute to the observed variability.

### Mesh type

Mesh material emerged as a key determinant of outcome. Subgroup analysis revealed a significant reduction in IH with synthetic mesh, whereas biologic mesh showed no clear benefit. Synthetic meshes, particularly polypropylene, offer durable mechanical support and consistent integration, while biologic meshes vary in resorption profiles and tissue incorporation. Differences in biologic mesh type (e.g., porcine acellular matrix vs. Surgisis Gold) and properties (resorption profiles and tissue integration), fixation technique, and surgical context (e.g., contaminated vs. clean fields) may explain the inconsistent results across studies using biologic materials.

### Contamination level

The surgical environment—clean versus contaminated—also influenced outcomes. While PMR showed benefit in clean fields, no significant effect was observed in contaminated settings. This may reflect differences in bacterial load, tissue quality, and wound healing dynamics. However, the limited number of studies in contaminated or emergency contexts (two RCT) restricts the strength of these conclusions.

### Follow-up duration and assessment

Follow-up strategies varied widely, ranging from clinical examination to imaging modalities such as ultrasound and CT, with durations spanning 6 months to 5 years. Imaging tends to detect more hernias than clinical evaluation, and longer follow-up captures late recurrences. Inconsistent follow-up methods and durations likely influenced reported IH rates and contributed to heterogeneity. Attrition bias was generally low, but loss to follow-up was variably reported.

### Subgroup analyses, heterogeneity, and limitations

Despite conducting subgroup analyses by surgical indication, mesh type, BMI, and contamination status, substantial heterogeneity persisted across most comparisons. Many subgroups were represented by only one or two studies, limiting statistical power and precision, and reducing the reliability of subgroup-specific conclusions.

While high heterogeneity (as indicated by elevated I^2^ values) reflects variability in effect estimates across studies, it does not inherently invalidate a statistically significant pooled result. While the high heterogeneity prevents confident generalizability of the observed results on a given context, it is also evidence of a robust underlying signal that withstands confounding influences. Nonetheless, such findings must be interpreted with caution, as heterogeneity can obscure the consistency and generalizability of the effect across different clinical contexts. Further research is needed to elucidate the sources of heterogeneity and confirm the reliability of the observed benefit. Given these limitations, the subgroup findings should be considered exploratory rather than definitive.

### Secondary outcomes

No significant increase in surgical site infections was observed with PMR. However, non-significant trends toward increased seroma (OR 1.97) and hematoma formation (OR 3.05) warrant clinical attention, particularly in frail or immunocompromised patients. Mesh explantation was rare but did occur, underscoring the need for careful patient selection, as such a complication may offset any mesh-related benefit. Operative time was modestly increased in the mesh group, which may be relevant in emergency or unstable patients.

### Clinical implications

Identifying patients who benefit most from PMR remains a key challenge. Obesity is a well-established risk factor for IH, and several included studies targeted high-BMI populations. Although subgroup analysis did not show a statistically significant difference between obese and non-obese patients, point estimates suggest a possible benefit. Technical challenges for the placement of a mesh and increased wound-related complications in very high BMI patients highlight the importance of individualized risk–benefit assessment.

## Conclusion

Retromuscular PMR appears to significantly reduce the incidence of incisional hernia following elective midline laparotomy, particularly when synthetic mesh is used. Importantly, this benefit does not come at the cost of increased surgical site infections, although trends toward higher rates of seroma and hematoma formation warrant clinical attention. However, the substantial heterogeneity across studies, coupled with limited data in contaminated or emergency settings and with biologic meshes, underscores the need for cautious interpretation. These findings support the selective use of retromuscular PMR in high-risk elective cases, while highlighting the need for further high-quality randomized trials to clarify its role in emergency and contaminated surgical contexts.

## Supplementary Information

Below is the link to the electronic supplementary material.

Supplementary file1 (PDF 269 KB)

Supplementary file2 (PDF 118 KB)

Supplementary file3 (DOCX 27 KB)

## Data Availability

The datasets during and/or analysed during the current study available from the corresponding author on reasonable request.
