# Correction to: Retromuscular prophylactic mesh reinforcement after midline laparotomy: a systematic review and meta-analysis

**DOI:** 10.1007/s10029-026-03791-8

**Published:** 2026-07-09

**Authors:** Melissa Lagger, Raffaella Sguinzi, Leo Buhler, Michel Adamina

**Affiliations:** 1 Department of Surgery, Cantonal Hospital of Fribourg, Chemin Des Pensionnats 2/6, 1752 Villars-Sur-Glâne, Fribourg Switzerland; 2 Department of Medical and Surgical Specialties, Faculty of Science and Medicine, University of Fribourg, Fribourg, Switzerland; 3 Department of Visceral Surgery and Medicine, Inselspital, Bern University Hospital, Bern, Switzerland

**Correction to: Hernia**

10.1007/s10029-025-03533-2

In the original version of this article, the given and family names of authors Raffaella Sguinzi, Leo Buhler, Michel Adamina were incorrectly structured.

The original article has been corrected. 

