# Supplementary material for: Retromuscular prophylactic mesh reinforcement after midline laparotomy: a systematic review and meta-analysis
Source: Hernia. 2025 Dec 27;30(1):45. doi: 10.1007/s10029-025-03533-2 (PMC12743063; doi:10.1007/s10029-025-03533-2)
Supplement: Supplementary file 3 — Supplementary file3 (DOCX 27 KB) [file 10029_2025_3533_MOESM3_ESM.docx]

**Supplementary material**

| #1 | (incisional hernia OR hernia rates OR hernia prevention OR hernia prophylaxis) [tw] |
| --- | --- |
| #2 | Incisional hernia [mesh/emtree] |
| #3 | #1 OR #2 |
| #4 | (elective laparotomy OR emergency laparotomy OR median laparotomy OR midline abdominal incision OR open abdominal surgery OR bariatric surgery) [tw] |
| #5 | Laparotomy [ mesh/emtree ] |
| #6 | #4 OR #5 |
| #7 | (Retromuscular mesh OR Sublay mesh OR Prophylac* mesh OR mesh reinforcement OR retrorectus mesh) [tw] |
| #8 | Surgical mesh [mesh/emtree ] |
| #9 | #7 OR #8 |
| #10 | #3 AND #6 AND #9 |

**Table 1** Search strategy
